# Prognostic Value of QRS Duration in Patients with Dilated Cardiomyopathy According to Left Ventricular Ejection Fraction

**DOI:** 10.31083/j.rcm2412362

**Published:** 2023-12-25

**Authors:** Jiayu Feng, Xuemei Zhao, Boping Huang, Yihang Wu, Jing Wang, Jingyuan Guan, Liyan Huang, Xinqing Li, Yuhui Zhang, Jian Zhang

**Affiliations:** ^1^State Key Laboratory of Cardiovascular Disease, National Center for Cardiovascular Diseases, Fuwai Hospital, Chinese Academy of Medical Sciences and Peking Union Medical College, 10000 Beijing, China; ^2^Key Laboratory of Clinical Research for Cardiovascular Medications, National Health Committee, 10000 Beijing, China

**Keywords:** dilated cardiomyopathy, cardiac resynchronization therapy, prognosis, electrocardiography

## Abstract

**Background:**

The prognostic significance of QRS duration (QRSd) in patients with dilated cardiomyopathy (DCM) and a left ventricular ejection fraction (LVEF) between 30% and 50% is unclear, resulting in questions regarding eligibility for cardiac resynchronisation therapy. This study aimed to explore the prognostic role of QRSd in patients with DCM and a LVEF 30–50% or LVEF <30%

**Methods:**

Patients hospitalised at Fuwai hospital with DCM who had a LVEF ≤50% were prospectively included. The primary outcomes were a composite of death, heart transplantation, and rehospitalisation for worsening heart failure.

**Results:**

Among the 633 patients included, 302 (47.7%) had a LVEF of 30–50%. The multivariable hazard ratio (HR) for QRSd ≥120 ms was 1.65 (95% confidence interval [CI] 1.29–2.11, *p* < 0.001) for overall DCM patients, 2.8 (95% CI 1.82–4.30, *p* < 0.001) for patients with LVEF 30–50%, and 1.41 (95% CI 1.02–1.94, *p* = 0.036) for patients with LVEF <30%. QRSd ≥120 ms tended to be more strongly associated with outcome in patients with LVEF 30–50% than in those with LVEF <30% despite the non-significant interaction (*p* = 0.067). DCM patients with QRSd ≥120 ms and LVEF 30–50% did not experience a significantly better outcome than those with LVEF <30% and QRSd <120 ms after propensity-score matching (HR 0.91, 95% CI 0.61–1.36, *p* = 0.645).

**Conclusions:**

QRSd independently predicts prognosis in DCM patients irrespective of LVEF and identifies a group of high-risk patients who may benefit from device implantation despite the absence of severely reduced LVEF.

## 1. Introduction 

Dilated cardiomyopathy (DCM) is a cardiac disorder characterised by ventricular dilation and systolic dysfunction that may lead to adverse cardiac events including heart failure (HF) and arrhythmias [1]. The current risk stratification for DCM is primarily based on the severity of left ventricular systolic dysfunction (LVSD), with a left ventricular ejection fraction (LVEF) of 30% or 35% as the threshold, which is also used to determine eligibility for device treatment such as cardiac resynchronisation therapy (CRT) or implantable cardioverter-defibrillator (ICD) [2]. Nevertheless, relatively few patients with LVEF of 30–50% have been randomised into trials to receive device therapy. Consequently, these patients lack valuable markers for predicting outcomes and determining whether they require device treatment.

An earlier study found that patients with mild-to-moderate cardiomyopathy (ischaemic or non-ischaemic, LVEF 36–50%) who had complicated diabetes mellitus (DM) were at greater risk of poor prognosis than severe cardiomyopathy patients without DM (LVEF ≤35%) [3]. However, in patients with non-ischaemic DCM and an LVEF of 30–50%, relevant risk factors determining the best beneficiaries of therapy remain undefined. Several studies have shown the prognostic role of prolonged QRS duration (QRSd) in heart failure with reduced ejection fraction (HFrEF) and heart failure with preserved ejection fraction (HFpEF), supporting its use as a risk stratification tool [4, 5, 6]. A previous study demonstrated that characteristics including age, male gender, history of DCM and reduced LVEF were independently associated with QRSd ≥120 ms [4]. However, evidence regarding the prognostic role of prolonged QRSd remains limited in patients with non-ischaemic DCM stratified by LVEF, especially in those with an LVEF 30–50%.

Based on this, our research aims to (1) explore the prognostic effects of QRSd ≥120 ms in patients with DCM and a LVEF 30–50% or LVEF <30% and (2) examine the outcomes in patients with DCM and QRSd ≥120 ms and LVEF 30–50% versus those with QRSd <120 ms and LVEF <30% to improve the risk stratification for DCM and identify the appropriate patient population for device implantation.

## 2. Materials and Methods

### 2.1 Patients

We prospectively included patients admitted to the Fuwai Hospital between 2006 and 2017 with a diagnosis of DCM and an LVEF ≤50%. We excluded patients (1) with coronary heart disease (CAD) or other types of cardiomyopathies; (2) with LVEF >50%; (3) with a pacemaker, ICD, or CRT; (4) with missing electrocardiogram (ECG), echocardiography, or follow-up data; and (5) with an inconsistent diagnosis of left bundle branch block (LBBB) or QRS widths (LBBB present and QRSd <120 ms).

### 2.2 Data Collection and Outcomes

Patients with DCM were further stratified into groups with LVEF 30–50% or LVEF <30%. Demographic, diagnostic, laboratory test, medical therapy, ECG, and echocardiography data were obtained from an electronic medical system. A 2-dimensional echocardiogram was performed by an imaging expert, and LVEF was calculated using the Simpson method with apical 2- and 4-chamber views. The QRSd was obtained from automatic ECG readings and confirmed by a cardiologist.

The primary outcome was a composite of death, heart transplantation, and first-time readmission owing to worsening HF. Follow-up was conducted through clinic visits or telephone calls after discharge. All participants signed an informed consent form, and the study was conducted in accordance with the Declaration of Helsinki with the approval of the ethics committee.

### 2.3 Statistical Analysis

We performed statistical analyses to compare the characteristics of patients with QRSd <120 ms and QRSd ≥120 ms. Categorical variables were assessed using the χ^2^ test, whereas continuous variables were evaluated using the Mann-Whitney U test. Additionally, multivariate logistic regression was employed to determine the characteristics that were independently correlated with QRSd ≥120 ms.

We used the Kaplan-Meier curves and log-rank tests to compare the outcomes in the LVEF 30–50% group vs. LVEF <30% group, the QRSd <120 ms group vs. QRSd ≥120 ms group, and among the four-level groups (LVEF 30–50% vs. LVEF <30%, and QRSd ≥120 ms vs. QRSd <120 ms). Cox regression analyses were performed to investigate the independent prognostic role of QRS prolongation in the overall LVEF 30–50%, and LVEF <30% cohorts. Variables routinely available in clinics and known to be associated with prognosis were selected prospectively, including age, sex, history of hypertension, history of atrial fibrillation (AF), history of diabetes, New York Heart Association (NYHA) class, haemoglobin levels, log-transformed creatine levels, therapy with angiotensin converting enzyme inhibitor/angiotensin receptor blocker (ACEI/ARB), and β-blockers, to establish two baseline models (including log-transformed N-terminal pro brain natriuretic peptide (NT-ProBNP) or untransformed). Restricted cubic splines (using 4 knots) were used to investigate the potential non-linear relationship between QRSd and outcomes. After that, a 1:1 propensity-score-matched cohort for age, sex, history of hypertension and left ventricular end-diastolic diameter between patients with QRSd ≥120 ms and LVEF 30–50%, and those with QRSd <120 ms and LVEF <30% was constructed, and the outcome of these two groups were compared. Schoenfeld residual plots were used to test the proportional hazard assumption.

For the sensitivity analysis, we also performed the above analysis in patients without LBBB. Moreover, we evaluated the discriminative ability of the best prediction model by adding QRS prolongation to predict the composite outcome using Harrell’s C-statistic. The variables in the best model were based on stepwise selection and important factors (age and sex) with a significance level of 0.1 for entry and retention. Finally, net reclassification improvement (NRI) and integrated discrimination improvement (IDI) were assessed over five years. Statistical significance was defined as a *p*-value < 0.05. Statistical analyses were conducted using R software version 4.1.3 (R Foundation for Statistical Computing, Vienna, Austria).

## 3. Results

### 3.1 Baseline Characteristics and Predictors of QRSd ≥120 ms

**Fig. 1.**
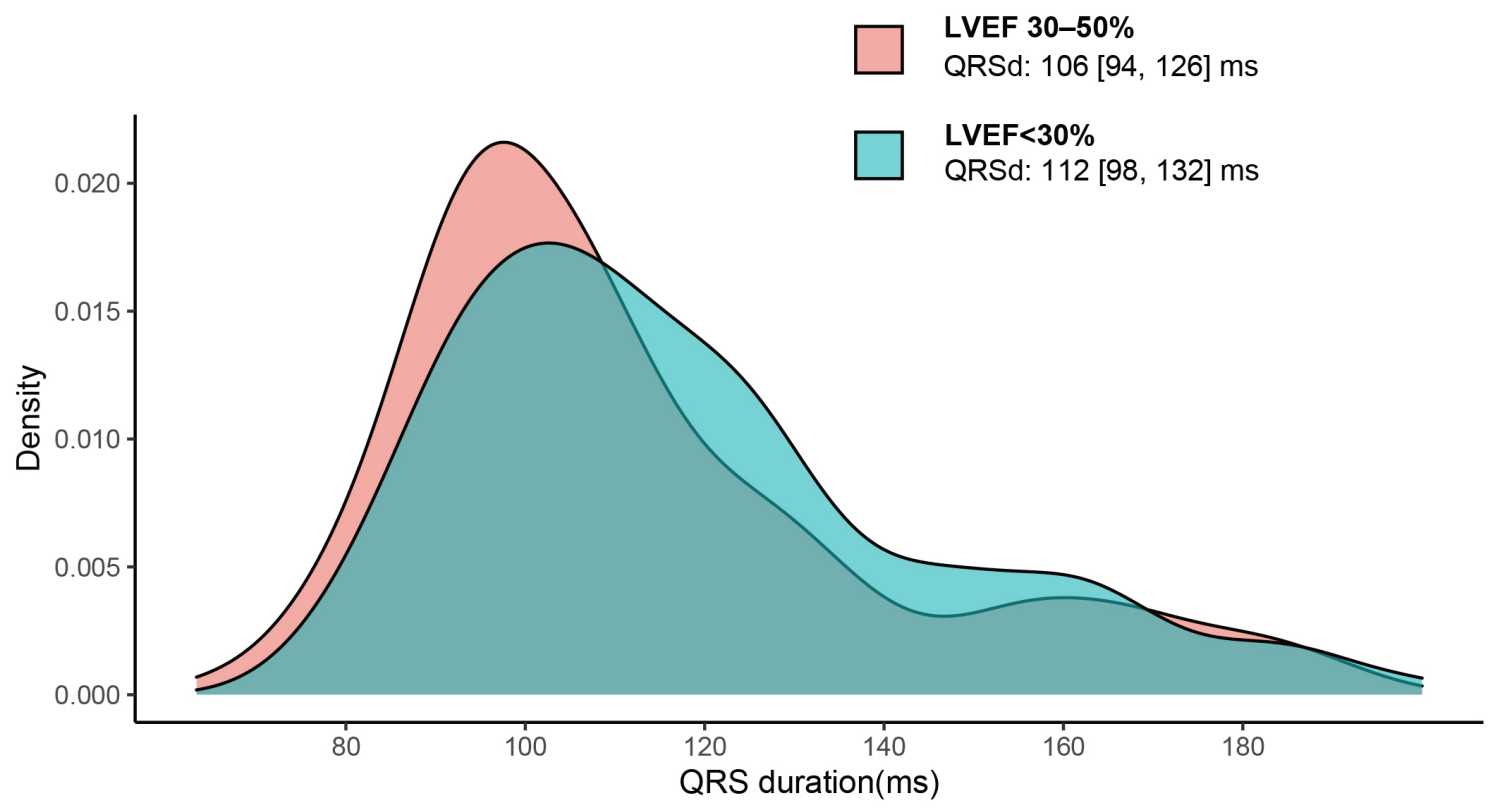
**QRS duration distribution for DCM patients with LVEF 30–50% and LVEF <30%**. The median (IQR) of QRSd was 106 (94, 126) ms in patients with LVEF 30–50% and 112 (98, 132) in patients with LVEF <30%, *p* = 0.003. DCM, dilated cardiomyopathy; LVEF, left ventricular ejection fraction; QRSd, QRS duration.

We included 633 patients with DCM in this study, of which 47.7% of the patients had a LVEF 30–50% and 35.7% of the patients had QRSd ≥120 ms (**Supplementary Fig. 1**). The distribution of QRSd in the groups with LVEF 30–50% and LVEF <30% is shown in Fig. 1. A comparison of characteristics between patients with QRSd ≥120 ms and those with QRSd <120 ms is shown in Table 1. **Supplementary Table 1** presents the characteristics stratified by LVSD severity, whereas** Supplementary Table 2** displays the characteristics categorised into four groups. Patients with QRSd ≥120 ms had lower systolic blood pressure (SBP), a reduced body mass index, higher NT-ProBNP level, and lower LVEF than those with QRSd <120 ms, the usage of ACEI/ARBs and β-blockers were also lower among these patients. Characteristics including age (odds ratio [OR] 1.03), heart rate (OR 0.99), LVEF (OR 0.97), and history of diabetes (OR 0.56) were independently associated with QRSd ≥120 ms (*p* < 0.05, **Supplementary Table 3**).

**Table 1.** **Baseline characteristics for patients with DCM and QRS duration <120 ms vs. ≥120 ms**.

N		Overall	QRSd <120 ms	QRSd ≥120 ms	*p*-value
		633	407	226	
Clinical characteristics					
	Age (years)	48 [36, 59]	46 [32, 57]	52 [43, 62]	<0.001
	Female (%)	151 (23.9)	94 (23.1)	57 (25.2)	0.614
	Heart rate (b.p.m)	83 [72, 96]	86 [75, 98]	79 [70.25, 92]	<0.001
	SBP (mmHg)	112 [100, 124]	113 [101.50, 125]	110 [98, 120]	0.003
	DBP (mmHg)	71 [63, 80]	73 [65, 82]	70 [60, 75.75]	<0.001
	BMI (kg/$m^{2}$)	24.28 [21.48, 27.48]	24.71 [21.92, 27.81]	23.33 [20.41, 26.55]	0.001
	Diabetes (%)	109 (17.2)	78 (19.2)	31 (13.7)	0.103
	Hypertension (%)	186 (29.4)	121 (29.7)	65 (28.8)	0.869
	NYHA Class III/IV (%)	505 (79.8)	315 (77.4)	190 (84.1)	0.057
	Smoking (%)	200 (50.1)	129 (49.2)	71 (51.8)	0.700
	Length of stay (days)	10 [8, 14]	10 [8, 13]	11 [8, 14]	0.002
Electrocardiography					
	QRS duration (ms)	108 [96, 128]	100 [92, 108]	144 [128, 164]	<0.001
	PR interval (ms)	176 [160, 196]	174 [156.50, 189.92]	186 [164.35, 208]	<0.001
	QT interval (ms)	392 [362, 434]	380 [355, 412]	426.50 [390, 454.75]	<0.001
	QTc interval (ms)	457 [430, 486]	447 [423.76, 470.50]	481.50 [453, 505]	<0.001
	AF (%)	142 (22.4)	94 (23.1)	48 (21.2)	0.662
	NSVT (%)	172 (27.2)	102 (25.1)	70 (31.0)	0.131
Laboratory Test					
	Haemoglobin (g/L)	147 [134, 160]	148 [136, 161]	145 [131, 157]	0.039
	WBC (${\mathrm{10}}^{9}$/L)	7.22 [6.11, 8.64]	7.36 [6.16, 8.66]	6.97 [5.96, 8.55]	0.360
	K (mmol/L)	3.95 [3.67, 4.26]	3.91 [3.65, 4.23]	4.00 [3.74, 4.28]	0.047
	Na (mmol/L)	137.96 [135, 140]	138 [135.30, 140]	137.04 [134.49, 139.99]	0.083
	FBG (mmol/L)	5.06 [4.60, 5.76]	5.08 [4.61, 5.86]	4.99 [4.59, 5.61]	0.278
	Scr (umol/L)	90.05 [75.88, 107.05]	91.04 [75.33, 106.82]	88.90 [77.22, 107.78]	0.856
	NT-ProBNP (pg/mL)	2142 [953.50, 4886.65]	1984 [934, 4260]	2557 [1058, 5544]	0.050
Echocardiography					
	LAD (mm)	45 [41, 50]	45 [41, 50]	46 [41, 52]	0.028
	LVEDD (mm)	69 [63, 75]	68 [63, 73]	71 [64, 79.75]	<0.001
	LVEF (%)	29 [24, 34]	30 [24, 35]	28 [23, 33]	0.009
	RVD (mm)	25 [22, 29]	25 [22, 29]	25 [22, 28]	0.317
Therapy					
	Digoxin (%)	512 (80.9)	333 (81.8)	179 (79.2)	0.486
	ACEI/ARB (%)	453 (71.6)	304 (74.7)	149 (65.9)	0.024
	β-blocker (%)	580 (91.6)	380 (93.4)	200 (88.5)	0.049
	MRA (%)	584 (92.3)	380 (93.4)	204 (90.3)	0.214
	Diuretics (%)	515 (81.4)	329 (80.8)	186 (82.3)	0.729

The values are presented as the median [interquartile range] or as frequencies with corresponding percentages. SBP, systolic blood pressure; DBP, diastolic blood pressure; BMI, body mass index; AF, atrial fibrillation; NSVT, non-sustained ventricular tachycardia; LAD, left atrial diameter; LVEDD, left ventricular end-diastolic diameter; LVEF, left ventricular ejection fraction; RVD, right ventricular diameter; WBC, white blood cell; Scr, serum creatine; NT-ProBNP, N-terminal pro brain natriuretic peptide; ACEI, angiotensin converting enzyme inhibitor; ARB, angiotensin receptor blocker; MRA, mineralocorticoid receptor antagonists; DCM, dilated cardiomyopathy; QRSd, QRS duration; NYHA, New York Heart Association; FBG, fasting blood glucose.

### 3.2 A Comparison of Primary Outcome for DCM Patients with LVEF 30–50% vs. LVEF <30%

**Fig. 2.**
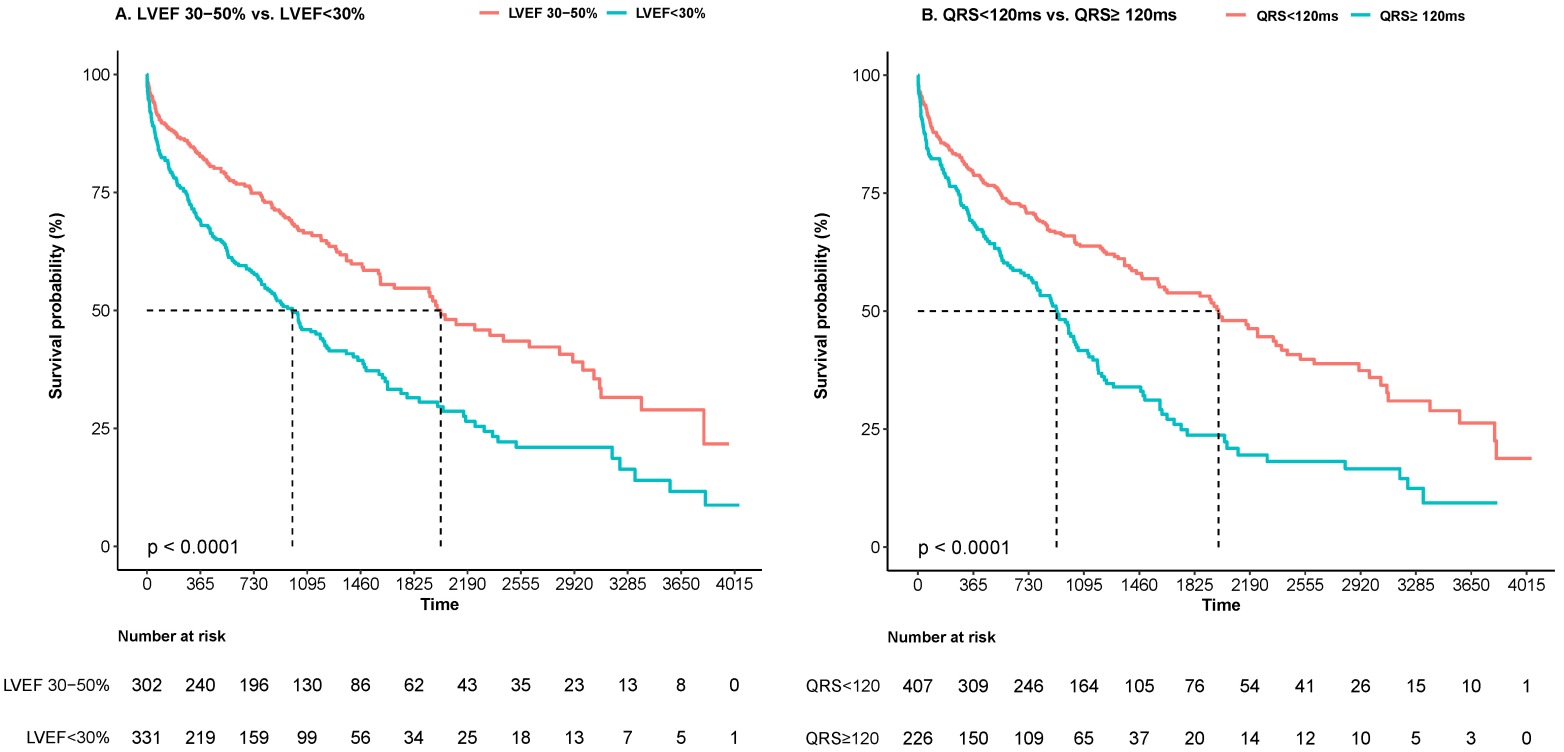
**Comparison of the primary outcome between patients with LVEF 30–50% and LVEF <30% (A), and between patients with QRSd <120 ms vs. QRSd ≥120 ms (B)**. LVEF, left ventricular ejection fraction; QRSd, QRS duration.

During a median follow-up of 33 (12–53) months, one of the primary outcomes occurred in 331 patients, of whom 192 died (30.3%), 26 underwent heart transplantation (4.1%), and 113 were readmitted for worsening HF (17.9%). The event rates separated by LVSD and QRS groups are shown in **Supplementary Table 4**. Patients with DCM and a LVEF <30% had worse outcomes than those with LVEF 30–50% (crude HR 1.81, 95% CI 1.45–2.25, *p* < 0.001). Kaplan-Meier curves are presented in Fig. 2A. Patients with LVEF <30% still had a higher risk of adverse events after adjusting for confounders (adjusted HR 1.38, 95% CI 1.09–1.76, *p* = 0.009).

### 3.3 Prognostic Value of QRSd in Overall DCM Patients and When Stratified by LVEF

In the overall cohort, patients with QRSd ≥120 ms exhibited a higher likelihood of reaching one of the primary endpoints than the QRSd <120 ms group (crude HR 1.86, 95% CI 1.49–2.31, *p* < 0.001, Fig. 2B). As a continuous variable, increasing QRSd was also associated with outcomes (crude HR 1.07, 95% CI 1.04–1.09, *p* < 0.001, per 10 ms increase). After controlling for potential confounding factors with NT-ProBNP, this association was observed both when considering it as a categorical variable (adjusted HR 1.65, 95% CI 1.29–2.11, *p* < 0.001) and as a continuous variable (adjusted HR 1.06, 95% CI 1.03–1.09, *p* < 0.001 per 10 ms increase).

**Fig. 3.**
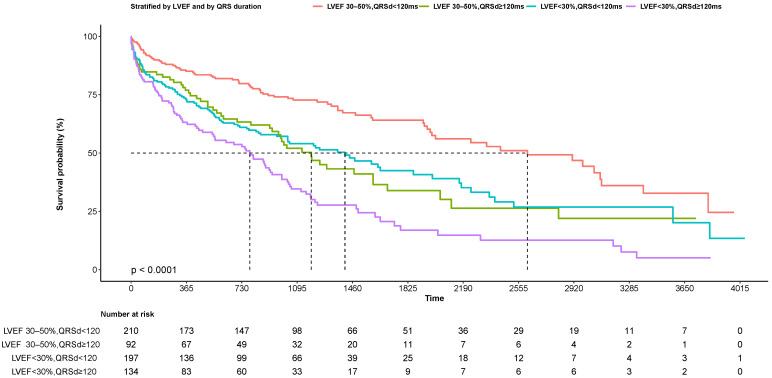
**Kaplan-Meier survival curves of patients with DCM stratified by LVEF and by QRS duration**. DCM, dilated cardiomyopathy; LVEF, left ventricular ejection fraction; QRSd, QRS duration.

A QRSd ≥120 ms was also an independent predictor for the composite outcome when patients were stratified by LVEF (Table 2). QRSd ≥120 ms showed prognostic value in patients with DCM and a LVEF 30–50%, the unadjusted HR was 1.95 (95% CI 1.37–2.78, *p* < 0.001) and the adjusted HR was 2.8 (95% CI 1.82–4.30, *p* < 0.001). The result was consistent in patients with DCM who had a LVEF <30%, with the crude HR 1.63 (95% CI 1.23–2.16, *p* < 0.001) and the adjusted HR 1.41 (95% CI 1.02–1.94, *p* = 0.036). However, the prognostic role of increasing QRS as a continuous variable (per 10 ms) was significant in patients with LVEF 30–50% (adjusted HR 1.06, 95% CI 1.03–1.10, *p* < 0.001) but not in the LVEF <30% group (adjusted HR 1.04, 95% CI 0.99–1.10, *p* = 0.116) in a multivariate model including NT-ProBNP. There was no statistically significant interaction between LVEF and QRSd as a binary (*p* = 0.067) or continuous (*p* = 0.975) variable. Restricted cubic splines of the association between QRSd and the outcomes are shown in **Supplementary Fig. 2**. We also used a 4-level variable (LVEF 30–50% vs. LVEF <30%, and QRSd ≥120 ms vs. <120 ms) to compare the outcome among four groups (Fig. 3). Patients with a QRSd ≥120 ms and a LVEF 30–50% had similar event-free survival to those who had a LVEF <30% and QRSd <120 ms (HR 0.94, 95% CI 0.68–1.32, *p = *0.73). In addition, propensity-score matching was conducted between patients with a LVEF 30–50% and QRSd ≥120 ms and those with a LVEF <30% with QRSd <120 ms for age, sex, history of hypertension, and left ventricular end-diastolic diameter. The results of the overall cohort were consistent with those in the matching cohort (HR 0.91, 95% CI 0.61–1.36, *p* = 0.645; **Supplementary Fig. 3**).

**Table 2.** **Prognostic value of QRS duration in overall DCM cohort, patients with LVEF 30–50% and LVEF <30%**.

Populations	Model	HR (95% CI)	*p*-value	HR (95% CI)	*p*-value
		QRSd ≥120 vs. <120 ms		QRSd per 10 ms increase	
Overall cohort with DCM	Unadjusted	1.86 (1.49, 2.31)	<0.001	1.07 (1.04, 1.09)	<0.001
	Clinical Model	1.68 (1.33, 2.13)	<0.001	1.06 (1.03, 1.09)	<0.001
	Clincal Model+NT-ProBNP	1.65 (1.29, 2.11)	<0.001	1.06 (1.03, 1.09)	<0.001
LVEF 30–50%	Unadjusted	1.95 (1.37, 2.78)	<0.001	1.07 (1.03, 1.11)	<0.001
	Clinical Model	2.41 (1.61, 3.62)	<0.001	1.06 (1.02, 1.10)	0.002
	Clincal Model+NT-ProBNP	2.80 (1.82, 4.30)	<0.001	1.06 (1.03, 1.10)	<0.001
LVEF <30%	Unadjusted	1.63 (1.23, 2.16)	<0.001	1.07 (1.02, 1.12)	0.005
	Clinical Model	1.52 (1.12, 2.07)	0.008	1.05 (1.003, 1.11)	0.039
	Clincal Model+NT-ProBNP	1.41 (1.02, 1.94)	0.036	1.04 (0.99, 1.10)	0.116

The adjusted HR was calculated in multivariable COX regression model including age, gender, history of hypertension, history of atrial fibrillation, history of diabetes, NYHA class, hemoglobin, log-transformed creatine, therapy with ACEI/ARB and β-blockers, with and without log-transformed NT-ProBNP. DCM, dilated cardiomyopathy; LVEF, left ventricular ejection fraction; NT-ProBNP, N-terminal pro brain natriuretic peptide; ACEI, angiotensin converting enzyme inhibitor; ARB, angiotensin receptor blocker; HR, hazard ratio; QRSd, QRS duration; NYHA, New York Heart Association.

### 3.4 Sensitivity Analysis for Patients without LBBB

**Fig. 4.**
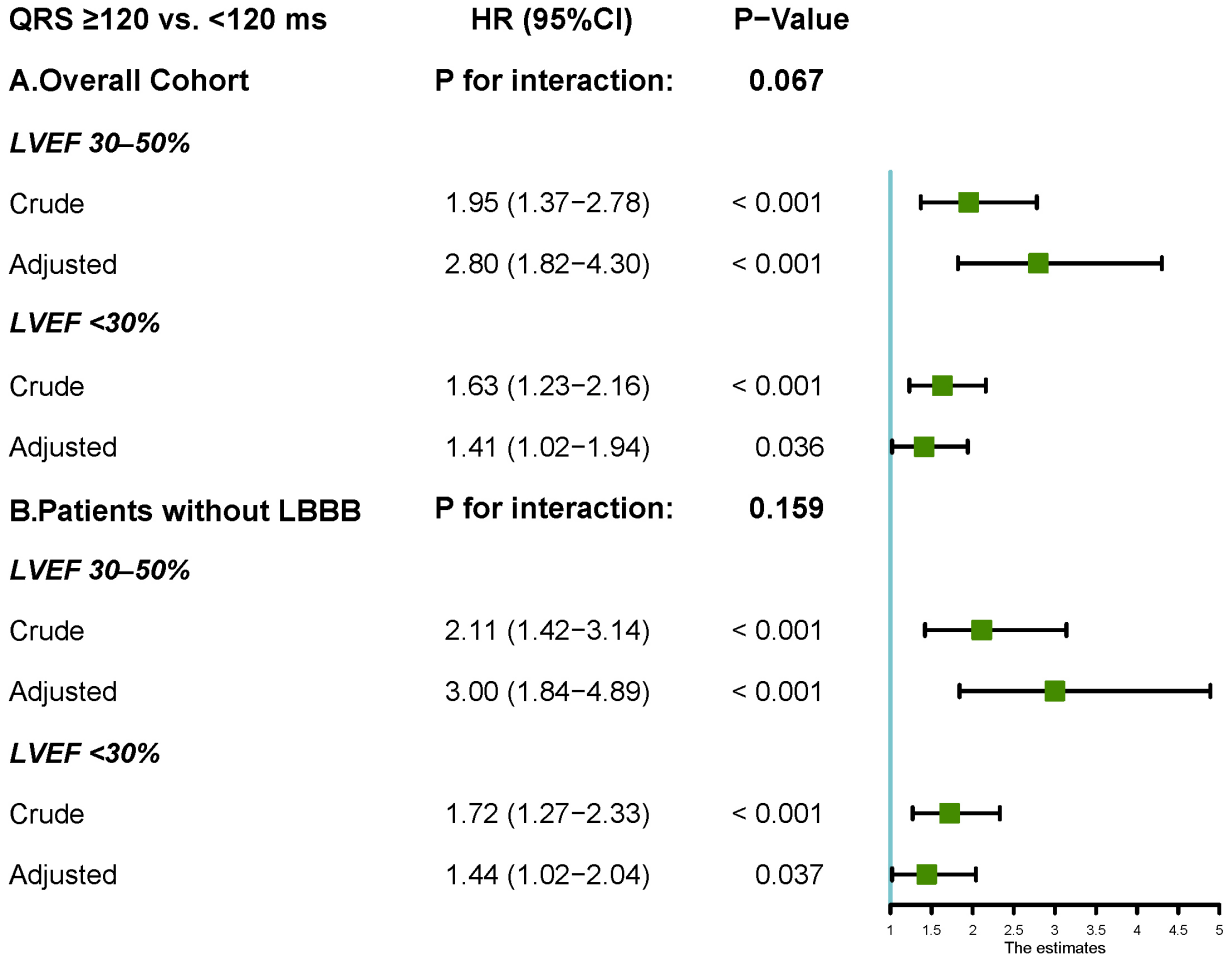
**Forest plots for the hazard ratios of QRS ≥120 ms stratified by LVEF in overall cohort and in patients without LBBB**. The adjusted HR was calculated in multivariable COX regression model including age, gender, history of hypertension, history of atrial fibrillation, history of diabetes, NYHA class, hemoglobin, log-transformed creatine, log-transformed NT-ProBNP, therapy with ACEI/ARB and β-blocker. LBBB, left bundle branch block; LVEF, left ventricular ejection fraction; NT-ProBNP, N-terminal pro brain natriuretic peptide; ACEI, angiotensin converting enzyme inhibitor; ARB, angiotensin receptor blocker; HR, hazard ratio; NYHA, New York Heart Association; HR, hazard ratio.

Sensitivity analyses were performed in 565 patients without LBBB. The prognostic value of QRS prolongation was consistent with that in the overall cohort and in patients without LBBB, when stratified by LVEF (Fig. 4). The multivariate HR for QRSd ≥120 ms was 1.69 (95% CI 1.29–2.21) in DCM patients without LBBB.

### 3.5 Discrimination and Reclassification of the Prediction Model Including QRS Prolongation

The best predictive model was determined using stepwise Cox regression (including age, sex, history of hypertension, history of AF, NYHA class, haemoglobin levels, sodium concentration, log-transformed NT-ProBNP, and therapy with ACEI/ARB and β-blockers) which yielded a c-index of 0.726 for the overall cohort of patients with DCM. QRSd ≥120 ms improved the c-statistics, IDI, and NRI for the overall population with DCM as well as for patients with a LVEF 30–50%, however, it did not achieve statistical significance for patients with LVEF <30% (Table 3).

**Table 3.** **Continuous net reclassification index (cNRI) and integrated discrimination improvement (IDI) index of the additional value of QRS prolongation of the prediction model**.

	ΔC-index	*p*-value	IDI	*p*-value	cNRI	*p*-value
Overall Cohort	0.006	<0.001	0.026 (0.007, 0.055)	0.002	0.219 (0.071, 0.326)	0.014
LVEF 30–50%	0.017	<0.001	0.071 (0.020, 0.127)	0.002	0.233 (0.041, 0.425)	0.020
LVEF <30%	0.003	0.052	0.016 (–0.003, 0.045)	0.138	0.226 (–0.109, 0.38)	0.154

The baseline model constructed based on stepwise regression [including age, gender, history of hypertension, history of atrial fibrillation, NYHA class, hemoglobin, Na, log-transformed NT-ProBNP, therapy with ACEI/ARB and β-blockers]. cNRI and IDI were calculated at 5 years follow-up. cNRI, continuous net reclassification index; IDI, integrated discrimination improvement; LVEF, left ventricular ejection fraction; NT-ProBNP, N-terminal pro brain natriuretic peptide; ACEI, angiotensin converting enzyme inhibitor; ARB, angiotensin receptor blocker; NYHA, New York Heart Association.

## 4. Discussion

### 4.1 Main Findings

In this study, 47.7% of the patients with DCM had a LVEF of 30–50%. Our results indicated that QRSd ≥120 ms was an independent predictor for outcome in the overall DCM patients, the patients with LVEF of 30–50%, and those with LVEF <30%. However, its independent prognostic role as a continuous variable (per 10 ms increase) was insignificant in patients with LVEF <30%. Moreover, DCM patients with QRSd ≥120 ms and LVEF of 30–50% experienced a similar prognosis to those with LVEF <30% and QRSd <120 ms.

### 4.2 LVSD in Patients with DCM

LVSD, determined by LVEF, remains the most critical parameter for diagnosis, phenotyping, and treatment decision-making in HF [7]. Patients with mild-to-moderately reduced LVEF (30% or 35%–50%), especially those with nonischaemic dilated cardiomyopathy, present with significant gaps in risk stratification and optimal treatment [8]. In this cohort of patients with DCM, nearly half had a LVEF of 30–50%. Despite having a better outcome than that in patients with an LVEF <30%, 20% of the patients died during follow-up. Although a previous DCM registry also confirmed that the risk is higher in patients with severely impaired LVEF, patients with mildly or moderately reduced LVEF are more common, and their risk remains significant [9]. Additionally, studies on patients with out-of-hospital cardiac arrest have shown that 70–80% have a LVEF >35%, suggesting that the majority of sudden cardiac death occur in patients with less severe LVSD [10, 11]. To further guide patients with an LVEF of 30–50%, it is important to identify the subset of this group of high-risk patients.

### 4.3 Prognostic Value of QRSd in DCM Patients across the Range of LVEFs

ECG has traditionally been considered nonspecific in DCM, but studies evaluating genotype-phenotype correlations have provided new insights into identifying specific abnormalities or subtypes of DCM [12]. Earlier studies found that QRS is an independent risk factor for all-cause death in patients with HF, regardless of age, sex, NYHA class, and LVEF (<30%, 30–39%, 40–49%, and >50%) [4, 13, 14, 15]. Furthermore, the presence of severe conduction disorders such as LBBB or prolonged QRSd not only increases the patient’s susceptibility to tachyarrhythmia but also elevates the risk of bradyarrhythmia with atrioventricular block, consequently reducing overall survival. Severely reduced LVEF results in a longer QRSd than mildly reduced LVEF owing to more severe remodelling and fibrosis [4]. In addition, Asians have a steeper increase in QRSd with a reduction in LVEF than whites [5]. We found similar findings in our study of a cohort of patients with nonischaemic DCM in China.

Moreover, DCM is more likely associated with prolonged QRS than ischaemic heart disease, suggesting that prolonged conduction is not the result of focal ischaemia [6, 16]. Based on the different relationships between the aetiologies and QRSd, we limited the study population to patients with DCM and LVEF ≤50%. Although there was no statistically significant interaction between QRSd ≥120 ms and LVEF in our study (*p* = 0.067), the association between QRSd ≥120 ms and composite events appeared to be more pronounced in patients with LVEF of 30–50% than in those with LVEF <30% (Fig. 4). The cause of the difference between the subgroups remains unclear, partly due to patients with low LVEF already being identified as a high-risk group. However, this suggests that patients with DCM and a QRSd ≥120 ms are at high risk of adverse events despite the absence of severe LVSD and need further risk stratification. The present data also show that the addition of QRS prolongation can improve model discrimination and reclassification, thus helping resolve the problems of risk stratification based only on LVEF and the poor specificity of LVEF-based guidelines [9].

### 4.4 Device Implantation in Patients with QRS Prolongation and LVEF 30–50%

A recent cardiac magnetic resonance (CMR) study showed that additional prognostic stratification could be obtained by combining late gadolinium enhancement (LGE) and QRSd, which could improve the appropriate placement of ICDs in DCM patients [17]. However, this study did not analyse a subgroup of patients with mild-to-moderate LVSD. Another study showed that mid-wall LGE identifies a group of DCM patients with a LVEF ≥40% were at increased risk of sudden death, suggesting these patients might benefit from ICD implantation [18]. Further research is required to investigate the prognostic significance of the combination of LGE and QRSd in patients with DCM and mild-to-moderate LVSD, as well as the benefits of LGE and wide QRS for CRT with a defibrillator (CRT-D). Other risk factors that prove to be predictors for this group of patients, such as older age, history of DM, HF, or haemoglobin levels [3], can also be included to build a predictive model using CMR and ECG parameters.

CRT or CRT-D has been class I recommended in patients with HF in sinus rhythm with a QRSd ≥150 ms, LBBB, and LVEF ≤35% despite the optimal medical therapy [2]. However, its effects have not yet been established in patients with less severe LVSD. Only some post-hoc analyses have suggested that CRT might be effective in patients with more mildly decreased LV function (LVEF >30% or 35%) [8, 19, 20]. Our study demonstrated that DCM patients with QRSd ≥120 ms and LVEF 30–50% did not experience a significantly better outcomes than those with LVEF <30% and QRSd <120 ms. This finding suggested that patients with QRSd ≥120 ms might benefit from a comprehensive assessment for device implantation, even in the absence of severely reduced LVEF. Such an evaluation should encompass factors such as a family history of arrhythmic risks, the presence of CMR-LGE, and dynamic changes in cardiac structure and function subsequent to guideline directed medical therapy (GDMT). Furthermore, patients with LVEF of 30–50% may also necessitate intensive pharmacological interventions, such as sacubitril-valsartan or sodium-glucose cotransporter 2 (SGLT2) inhibitors, both of which may reverse remodelling [21, 22]. Subsequently, a close follow-up should be implemented and the eligibility for CRT should be re-evaluated based on the responsiveness to drug treatment.

### 4.5 Limitations

This study had several limitations. First, the left ventricular (LV) function of patients with DCM may undergo a dynamic change during follow-up; however, relatively few patients had available follow-up echocardiograms; therefore, it was not analysed in the present study. Second, the limited sample size, notably when patients were stratified by LVSD severity, is another limitation of the current study. Therefore, instead of using LVEF <35% recommended by the current guidelines to classify severe LVSD, we used an LVEF cut-off of 30% to ensure a sufficient sample size in each subgroup. Third, because this was an observational study, meaning some potential confounding factors could not be adjusted for using multivariate analyses.

## 5. Conclusions

A QRSd ≥120 ms was independently associated with outcomes in overall patients with DCM, as well as in those with LVEF of 30–50% or LVEF <30%. QRSd ≥120 ms more strongly predicts outcomes in patients with LVEF of 30–50% than in those with LVEF <30%. DCM patients with QRSd ≥120 ms and LVEF of 30–50% did not experience a significantly better outcome to those with LVEF <30% and QRSd <120 ms. These data imply that QRS prolongation could help in risk stratification of patients with DCM regardless of LVEF. Further prospective studies are needed to verify the benefits of CRT or CRT-D implantation in DCM patients with an LVEF of 30–50% and prolonged QRSd.

## Data Availability

The datasets used and/or analyzed during the current study are available from the corresponding author on reasonable request.

## Supplementary Material

Supplementary material associated with this article can be found, in the online version, at https://doi.org/10.31083/j.rcm2412362.
