# Supplementary material for: Prognostic Value of QRS Duration in Patients with Dilated Cardiomyopathy According to Left Ventricular Ejection Fraction
Source: Rev Cardiovasc Med. 2023 Dec 25;24(12):362. doi: 10.31083/j.rcm2412362 (PMC11272873; doi:10.31083/j.rcm2412362)
Supplement: Supplementary file 1 [file 2153-8174-24-12-362-s1.zip › 2153-8174-24-12-362-s1.docx]

Supplementary Table 1. Baseline characteristics for the overall cohort, DCM patients with LVEF 30-50% and LVEF <30%.

|  | **Overall** | **LVEF 30-50%** | **LVEF <30%** | ***p*-Value** |
| --- | --- | --- | --- | --- |
| N | 633 | 302 | 331 |  |
| **Clinical characteristics** |  |  |  |  |
| Age(years) | 48 [36, 59] | 52 [39, 61] | 46 [33, 56] | <0.001 |
| Female (%) | 151 (23.9) | 84 (27.8) | 67 (20.2) | 0.032 |
| Heart rate (b.p.m) | 83 [72, 96] | 81.50 [71, 94] | 85 [75, 98] | 0.014 |
| SBP (mmHg) | 112 [100, 124] | 116 [105, 128] | 108 [98, 120] | <0.001 |
| DBP (mmHg) | 71 [63, 80] | 71.50 [65, 81] | 71 [62, 80] | 0.109 |
| BMI (kg/m2) | 24.28 [21.48, 27.48] | 24.75 [22.07, 27.61] | 23.66 [20.91, 27.36] | 0.017 |
| Diabetes (%) | 109 (17.2) | 62 (20.5) | 47 (14.2) | 0.045 |
| Hypertension (%) | 186 (29.4) | 115 (38.1) | 71 (21.5) | <0.001 |
| NYHA Class III/IV (%) | 505 (79.8) | 226 (74.8) | 279 (84.3) | 0.004 |
| Smoking (%) | 200 (50.1) | 88 (47.1) | 112 (52.8) | 0.294 |
| Length of stay (days) | 10 [8, 14] | 10 [8, 13] | 11 [8, 14] | 0.102 |
| **Electrocardiography** |  |  |  |  |
| QRS duration (ms) | 108 [96, 128] | 106 [94, 126] | 112 [98, 132] | 0.003 |
| PR interval (ms) | 176 [160, 196] | 174 [156, 192] | 180 [163, 200] | 0.041 |
| QT interval (ms) | 392 [362, 434] | 396 [364, 435.75] | 390 [360, 429] | 0.17 |
| QTc interval (ms) | 457 [430, 486] | 456 [428.30, 485] | 457 [431, 489] | 0.369 |
| QRSd≥120ms (%) | 226 (35.7) | 92 (30.5) | 134 (40.5) | 0.011 |
| AF (%) | 142 (22.4) | 79 (26.2) | 63 (19.0) | 0.04 |
| LBBB (%) | 68 (10.7) | 29 (9.6) | 39 (11.8) | 0.45 |
| NSVT (%) | 172 (27.2) | 69 (22.8) | 103 (31.1) | 0.025 |
| **Laboratory Test** |  |  |  |  |
| Haemoglobin (g/L) | 147 [134, 160] | 147 [133, 159] | 148 [135, 161] | 0.586 |
| WBC (10^9/L) | 7.22 [6.11, 8.64] | 7 [5.94, 8.46] | 7.39 [6.28, 8.86] | 0.017 |
| K (mmol/L) | 3.95 [3.67, 4.26] | 3.93 [3.64, 4.27] | 3.96 [3.70, 4.24] | 0.27 |
| Na (mmol/L) | 138 [135, 140] | 138 [136, 140] | 137 [134, 140] | 0.012 |
| FBG (mmol/L) | 5.06 [4.60, 5.76] | 5.07 [4.64, 5.83] | 5.04 [4.54, 5.66] | 0.166 |
| Scr (umol/L) | 90.05 [75.88, 107.05] | 89.30 [74.97, 105.66] | 91 [77.06, 108.59] | 0.188 |
| NT-Pro BNP (pg/ml) | 2142 [953.50, 4886.65] | 1746.95 [736.35, 3495.05] | 2772.30 [1297, 5475] | <0.001 |
| **Echocardiography** |  |  |  |  |
| LAD (mm) | 45 [41, 50] | 44 [40, 49] | 47 [42, 51] | <0.001 |
| LVEDD (mm) | 69 [63, 75] | 65 [60, 71] | 71 [66, 78] | <0.001 |
| LVEF (%) | 29 [24, 34] | 35 [31, 38.90] | 24 [20, 26] | <0.001 |
| RVD (mm) | 25 [22, 29] | 24 [21, 28] | 26 [23, 30] | <0.001 |
| **Therapy** |  |  |  |  |
| Digoxin (%) | 512 (80.9) | 235 (77.8) | 277 (83.7) | 0.076 |
| ACEI/ARB (%) | 453 (71.6) | 231 (76.5) | 222 (67.1) | 0.011 |
| β-blocker (%) | 580 (91.6) | 283 (93.7) | 297 (89.7) | 0.096 |
| MRA (%) | 584 (92.3) | 285 (94.4) | 299 (90.3) | 0.08 |
| Diuretics (%) | 515 (81.4) | 247 (81.8) | 268 (81.0) | 0.871 |

Values are shown as median [interquartile range] or as frequencies [percentage];

DCM: dilated cardiomyopathy; SBP: systolic blood pressure; DBP: diastolic blood pressure; BMI: body mass index; NYHA, New York Heart Association; AF: atrial fibrillation; LBBB: left bundle branch block; NSVT: Non-sustained ventricular tachycardia; LAD: left atrial diameter; LVEDD: left ventricular end-diastolic diameter; LVEF: left ventricular ejection fraction; RVD: right ventricular diameter; WBC: white blood cell; Scr: serum creatine; NT-Pro BNP: N-terminal Pro Brain natriuretic peptide; ACEI: Angiotensin converting enzyme inhibitor; ARB: Angiotensin receptor blocker; MRA: mineralocorticoid receptor antagonists.

**Supplementary Table 2. Baseline characteristics for patients with DCM stratified by LVEF and by the QRS duration.**

|  | **LVEF 30-50%**  **QRS≤120ms** | **LVEF 30-50%**  **QRS>120ms** | **LVEF<30%**  **QRS≤120ms** | **LVEF<30%**  **QRS>120ms** | ***p*-value** |
| --- | --- | --- | --- | --- | --- |
| N | 210 | 92 | 197 | 134 |  |
| **Clinical characteristics** |  |  |  |  |  |
| Age(years) | 49 [35, 60] | 55 [46, 63] | 43 [30, 54] | 49 [40, 59.75] | <0.001 |
| Female (%) | 61 ( 29.0) | 23 ( 25.0) | 33 ( 16.8) | 34 ( 25.4) | 0.032 |
| Heart rate (b.p.m) | 84 [72, 95] | 76.50 [66.75, 89.25] | 89 [77, 100] | 80 [74, 94] | <0.001 |
| SBP (mmHg) | 117 [105, 129] | 116 [103.75, 125] | 108 [100, 122] | 104.50 [92.25, 116] | <0.001 |
| DBP (mmHg) | 74.50 [65, 82.75] | 70 [63.75, 76] | 73 [64, 80] | 69 [60, 75] | <0.001 |
| BMI (kg/m2) | 24.91 [22.34, 28.09] | 24.56 [21.66, 27.14] | 24.42 [21.63, 27.73] | 22.43 [20.25, 26.18] | <0.001 |
| Diabetes (%) | 47 ( 22.4) | 15 ( 16.3) | 31 ( 15.7) | 16 ( 11.9) | 0.075 |
| Hypertension (%) | 74 ( 35.2) | 41 ( 44.6) | 47 ( 23.9) | 24 ( 17.9) | <0.001 |
| NYHA Class III/IV (%) | 154 ( 73.3) | 72 ( 78.3) | 161 ( 81.7) | 118 ( 88.1) | 0.008 |
| Smoking (%) | 58 ( 43.0) | 30 ( 57.7) | 71 ( 55.9) | 41 ( 48.2) | 0.123 |
| Length of stay (days) | 10 [7.25, 13] | 11 [8, 14] | 10 [8, 13] | 12 [8, 14.75] | 0.009 |
| **Electrocardiography** |  |  |  |  |  |
| QRS duration (ms) | 98 [90.36, 106] | 146.50 [128, 168.03] | 102 [92.56, 110] | 140.50 [126, 161.75] | <0.001 |
| PR interval (ms) | 170 [153, 188] | 186 [162, 204.75] | 176 [160, 190] | 187 [167.50, 209.50] | <0.001 |
| QT interval (ms) | 382 [356, 416] | 429 [403.75, 456] | 378 [354, 404] | 420 [386.60, 454] | <0.001 |
| QTc interval (ms) | 447 [422.25, 471] | 483 [452.25, 501.50] | 448 [425, 469] | 479.97 [454.25, 508] | <0.001 |
| AF (%) | 58 ( 27.6) | 21 ( 22.8) | 36 ( 18.3) | 27 ( 20.1) | 0.132 |
| NSVT (%) | 45 ( 21.4) | 24 ( 26.1) | 57 ( 28.9) | 46 ( 34.3) | 0.062 |
| **Laboratory Test** |  |  |  |  |  |
| Haemoglobin (g/L) | 148 [134, 159] | 145 [130.50, 157.50] | 149 [137, 163] | 145 [131.25, 157] | 0.119 |
| WBC (10^9/L) | 7.07 [6, 8.45] | 6.91 [5.68, 8.50] | 7.60 [6.38, 8.89] | 6.99 [6.16, 8.52] | 0.043 |
| K (mmol/L) | 3.91 [3.62, 4.27] | 3.98 [3.71, 4.26] | 3.91 [3.66, 4.22] | 4.03 [3.80, 4.28] | 0.144 |
| Na (mmol/L) | 138.10 [135.95, 140.21] | 138.20 [135.93, 139.84] | 137.50 [134.97, 139.90] | 136.25 [134, 140] | 0.036 |
| FBG (mmol/L) | 5.07 [4.64, 6.03] | 4.98 [4.66, 5.69] | 5.08 [4.55, 5.79] | 5 [4.51, 5.53] | 0.404 |
| Scr (umol/L) | 89.50 [73.45, 104.90] | 89.25 [78.33, 107.74] | 92.81 [77.20, 108.80] | 88.65 [76.84, 108.18] | 0.367 |
| NT-Pro BNP (pg/ml) | 1780.20 [789.90, 3227] | 1570 [520, 4527] | 2256.80 [1209, 5050.50] | 3452.30 [1640.75, 6843] | <0.001 |
| **Echocardiography** |  |  |  |  |  |
| LAD (mm) | 44 [40, 49] | 44 [40, 50] | 46 [41, 50] | 47 [43, 55] | <0.001 |
| LVEDD (mm) | 65 [61, 69] | 67 [60, 74] | 70 [65, 76] | 75 [67, 82] | <0.001 |
| LVEF (%) | 34 [31, 39] | 35 [31, 38.08] | 24 [21, 26.30] | 24 [20, 26] | <0.001 |
| RVD (mm) | 24 [22, 28] | 24 [21, 27] | 26 [23, 30] | 25 [23, 29] | <0.001 |
| **Therapy** |  |  |  |  |  |
| Digoxin (%) | 167 ( 79.5) | 68 ( 73.9) | 166 ( 84.3) | 111 ( 82.8) | 0.177 |
| ACEI/ARB (%) | 163 ( 77.6) | 68 ( 73.9) | 141 ( 71.6) | 81 ( 60.4) | 0.007 |
| β-blocker (%) | 199 ( 94.8) | 84 ( 91.3) | 181 ( 91.9) | 116 ( 86.6) | 0.066 |
| MRA (%) | 200 ( 95.2) | 85 ( 92.4) | 180 ( 91.4) | 119 ( 88.8) | 0.167 |
| Diuretics (%) | 171 ( 81.4) | 76 ( 82.6) | 158 ( 80.2) | 110 ( 82.1) | 0.957 |

Values are shown as median [interquartile range] or as frequencies [percentage];

DCM: dilated cardiomyopathy; SBP: systolic blood pressure; DBP: diastolic blood pressure; BMI: body mass index; NYHA, New York Heart Association; AF: atrial fibrillation; LBBB: left bundle branch block; NSVT: Non-sustained ventricular tachycardia; LAD: left atrial diameter; LVEDD: left ventricular end-diastolic diameter; LVEF: left ventricular ejection fraction; RVD: right ventricular diameter; WBC: white blood cell; Scr: serum creatine; NT-Pro BNP: N-terminal Pro Brain natriuretic peptide; ACEI: Angiotensin converting enzyme inhibitor; ARB: Angiotensin receptor blocker; MRA: mineralocorticoid receptor antagonists.

**Supplementary Table 3. Logistic regressions for independent predictors of QRS ≥120 ms.**

|  | **Crude OR (95%CI)** | **Crude P-value** | **Adjusted OR (95%CI)** | **Adjusted *p*-Value** |
| --- | --- | --- | --- | --- |
| Gender: female | 1.22 (0.8,1.84) | 0.356 | 0.89 (0.53,1.5) | 0.665 |
| **Age** | **1.02 (1.01,1.04)** | **< 0.001** | **1.03 (1.01,1.04)** | **< 0.001** |
| BMI | 0.96 (0.93,1) | 0.065 | 1.007 (0.96,1.05) | 0.77 |
| NYHA: III/IV vs. I/II | 1.45 (0.91,2.3) | 0.118 | 1.31 (0.79,2.19) | 0.293 |
| **Heart rate** | **0.99 (0.98,1)** | **0.005** | **0.99 (0.97,1)** | **0.013** |
| History of hypertension | 1.04 (0.71,1.53) | 0.83 | 1.2 (0.78,1.86) | 0.402 |
| History of atrial fibrillation | 0.82 (0.53,1.27) | 0.384 | 0.76 (0.47,1.25) | 0.28 |
| **History of diabetes** | **0.7 (0.43,1.13)** | **0.145** | **0.56 (0.33,0.95)** | **0.027** |
| Haemoglobin | 0.99 (0.98,1.0002) | 0.055 | 0.99 (0.98,1.006) | 0.345 |
| Na | 0.96 (0.92,1) | 0.046 | 0.96 (0.92,1.01) | 0.141 |
| WBC | 0.97 (0.89,1.06) | 0.511 | 1.02 (0.92,1.13) | 0.742 |
| **LVEF** | **0.97 (0.95,1)** | **0.031** | **0.97 (0.94,1)** | **0.021** |
| RVD | 0.99 (0.96,1.03) | 0.662 | 0.99 (0.95,1.03) | 0.553 |
| log Cr | 1.004 (0.64,1.57) | 0.985 | 0.9 (0.53,1.53) | 0.701 |
| log NT-Pro-BNP | 1.08 (0.97,1.19) | 0.161 | 1.02 (0.89,1.16) | 0.798 |
| Therapy with ACEI/ARB | 0.67 (0.45,1) | 0.048 | 0.73 (0.46,1.15) | 0.175 |
| Therapy β-blocker | 0.56 (0.3,1.05) | 0.071 | 0.86 (0.43,1.74) | 0.681 |

WBC: white blood cell; LVEF: left ventricular ejection fraction; RVD: right ventricular diameter; Cr: creatine; NT-Pro-BNP: N-terminal Pro Brain natriuretic peptide; ACEI: Angiotensin converting enzyme inhibitor; ARB: Angiotensin receptor blocker.

**Supplementary Table 4. The events rates according to LVEF and QRS group.**

|  | **Overall** | **LVEF 30~50%** | **LVEF 30~50%** | **LVEF<30%** | **LVEF<30%** | ***p*-value** |
| --- | --- | --- | --- | --- | --- | --- |
|  |  | **QRS≤120ms** | **QRS>120ms** | **QRS≤120ms** | **QRS>120ms** |  |
| N | 633 | 210 | 92 | 197 | 134 |  |
| Primary outcome | 331(52.3) | 79(37.6) | 52(56.5) | 103(52.3) | 97(72.4) | <0.001 |
| All-cause mortality | 192(30.3) | 33(15.7) | 29(31.5) | 59(29.9) | 71(53.0) | <0.001 |
| Heart transplantation | 26(4.1) | 3(1.4) | 3(3.3) | 13(6.6) | 7(5.2) | 0.041 |
| Rehospitalization for worsening HF | 113(17.9) | 43(20.5) | 20(21.7) | 31(15.7) | 19(14.2) | 0.288 |

LVEF: left ventricular ejection fraction.


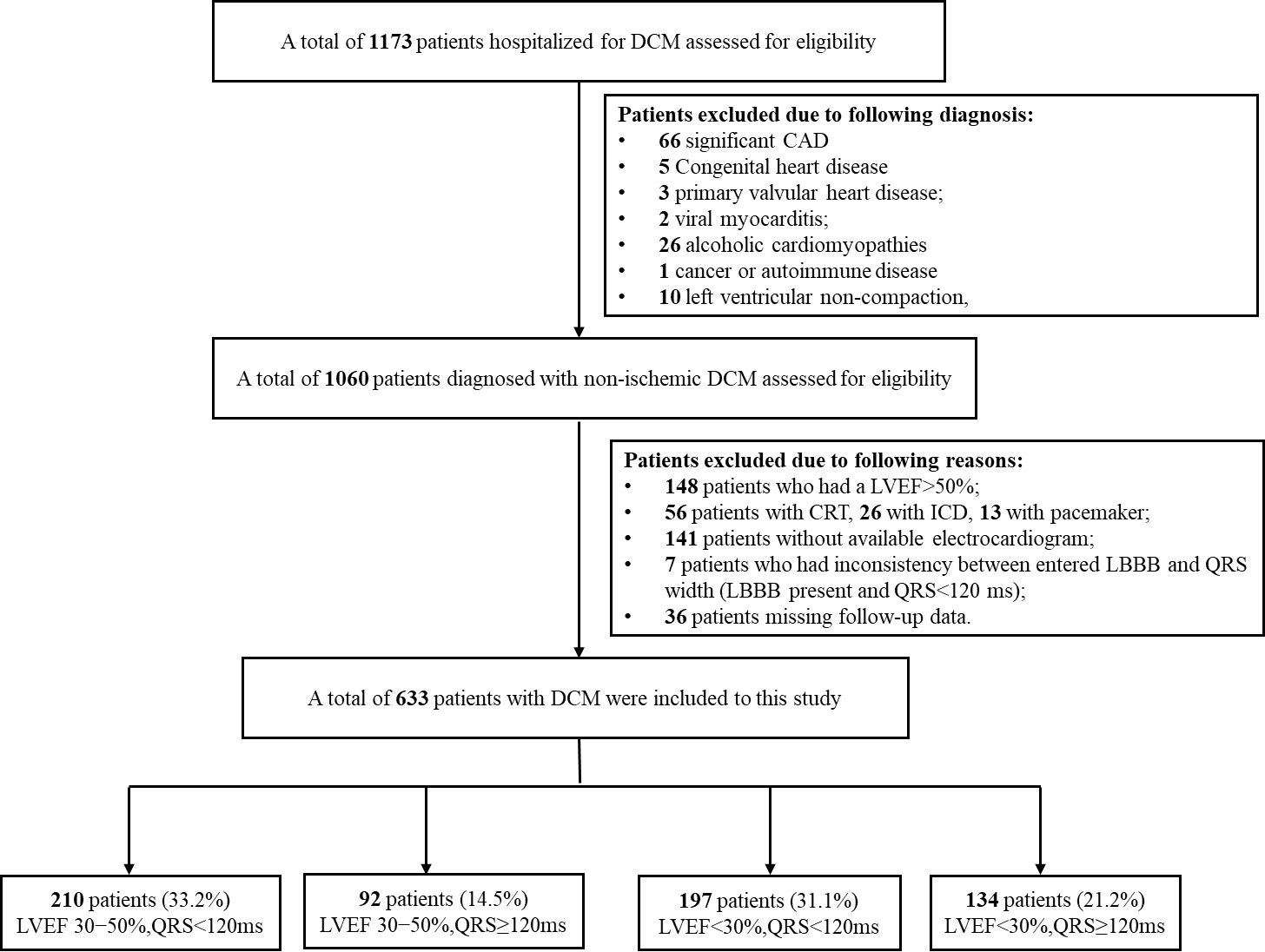


**Supplementary Fig. 1. Flowchart of the study.**

DCM: dilated cardiomyopathy; LVEF: left ventricular ejection fraction; CRT: cardiac resynchronization therapy; ICD: implantable cardioverter defibrillator; LBBB: left bundle branch block.


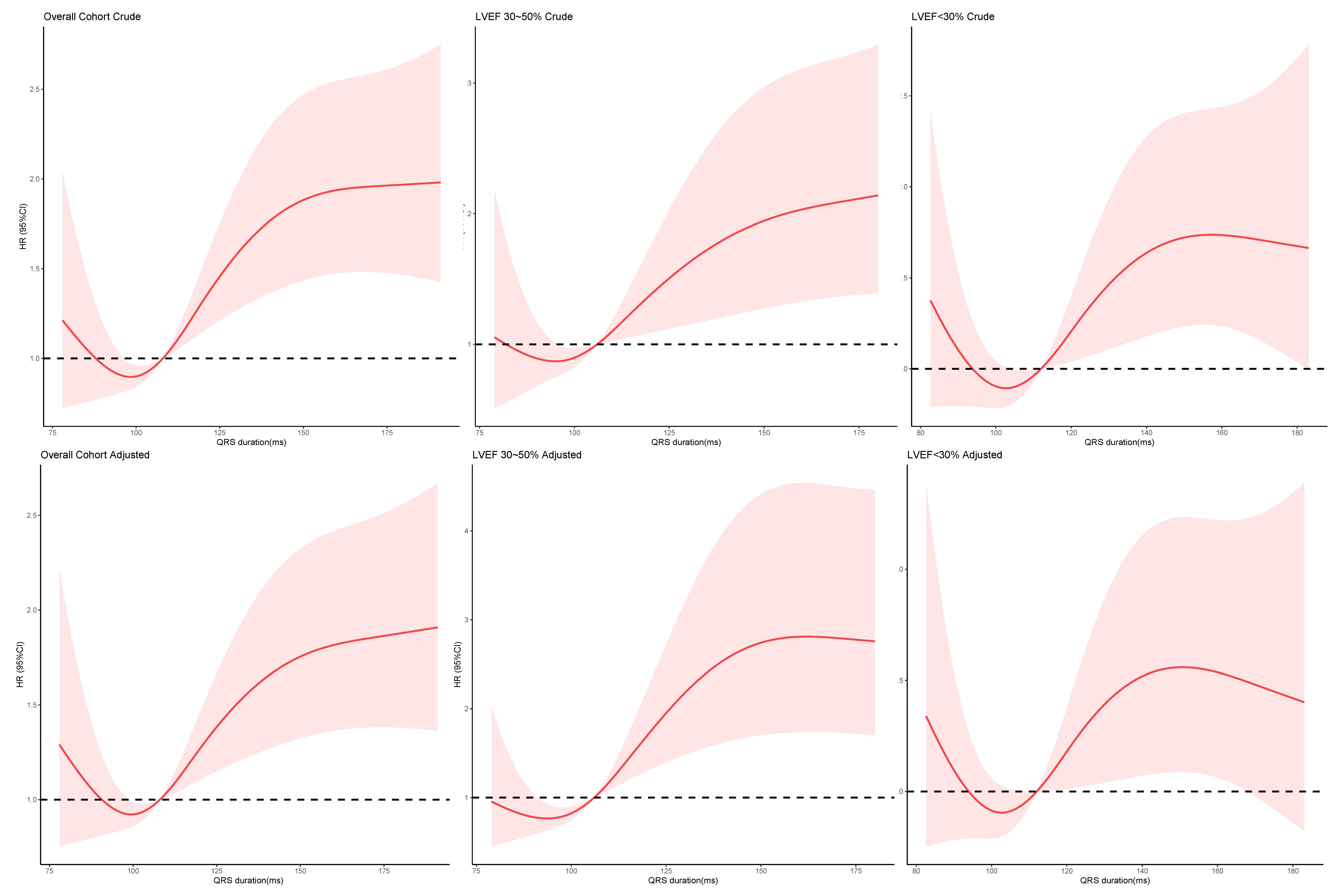


**Supplementary Fig. 2. Restricted cubic splines of the relationship between QRS duration measured as a continuous variable and outcome.**

The adjusted HR was calculated in multivariable COX regression model including age, gender, history of hypertension, history of atrial fibrillation, history of diabetes, NYHA class, hemoglobin, log-transformed creatine, log-transformed NT-Pro-BNP, therapy with ACEI/ARB and β-blocker.


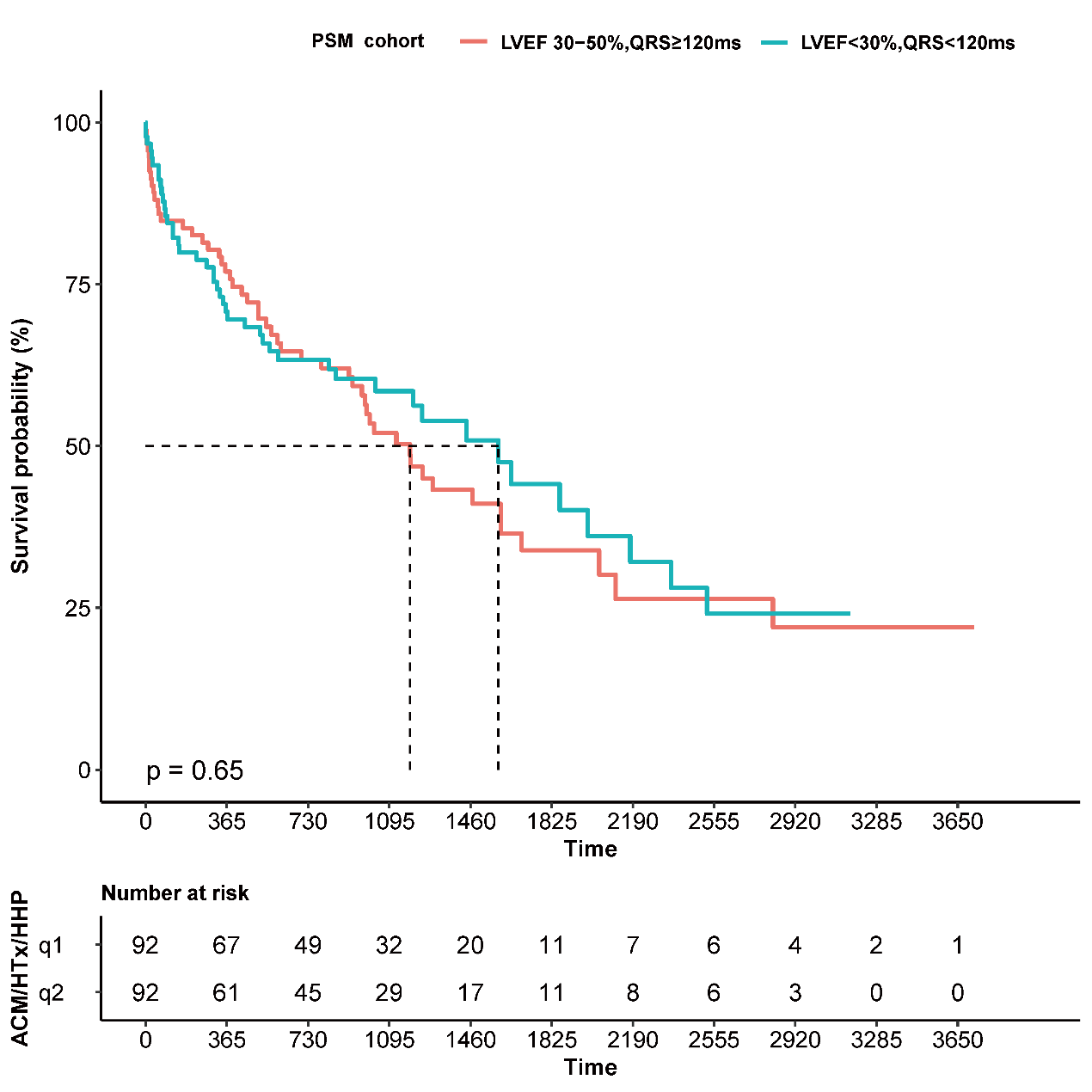


**Supplementary Fig. 3. Kaplan-Meier curves of the composite outcome in** **patients with LVEF 30~50% and QRSd ≥120ms, vs. those with LVEF <30% and QRSd <120ms, after propensity-score matching.**

Propensity-score matching was conducted between patients with LVEF 30~50% and QRSd ≥120ms, vs. those with LVEF <30% and QRSd <120ms for age, gender, history of hypertension and left ventricular end-diastolic diameter (LVEF 30-50% and QRS >120ms vs. LVEF<30% and QRS≤120ms: **HR 0.91, 95% CI 0.61-1.36, *p*=0.645**).
